# TURNING REAL-WORLD DATA INTO ACTIONABLE EVIDENCE FOR HEALTHCARE INTERVENTIONS IN NEURODEGENERATION

**DOI:** 10.1093/geroni/igac059.2224

**Published:** 2022-12-20

**Authors:** Rachel Poulsen, Jun Ha Chang, Danish Bhatti, Matthew Rizzo, Jennifer Merickel

**Affiliations:** University of Nebraska Medical Center, Omaha, Nebraska, United States; University of Nebraska Medical Center, Omaha, Nebraska, United States; University of Nebraska Medical Center, Omaha, Nebraska, United States; University of Nebraska Medical Center, Omaha, Nebraska, United States; University of Nebraska Medical Center, Omaha, Nebraska, United States

## Abstract

**Background:**

Age-related neurodegenerative disorders, including Alzheimer’s disease and Parkinson’s disease (PD), progressively reduce mobility and quality of life (QoL). Real-world mobility from actigraphy predicts PD disease severity. This pilot analysis assessed utility of actigraphy to screen early physical function-related QoL decline in PD. Method: Mobility was monitored for 4 weeks using wrist-worn ActiGraph recordings in 27 participants with idiopathic PD (age = 67.78 ± 5.64, 19 males). Days with >600 mins of wear time during non-sleep times were analyzed (µ = 29.78 days ± 3.78). Typical activity was quantified as average steps per hour. Participants completed demographic and health assessments. Disease severity and physical function QoL were measured using the clinically-validated Unified PD Rating Scale (MDS-UPDRS) and Short Form-36 (SF-36), respectively. Disease severity, QoL, and typical activity were compared using Spearman correlations.

**Results:**

Lower typical activity from actigraphy was associated with more severe motor symptoms (MDS-UPDRS; r = -0.40, p = 0.04) and with increased impairment in physical function QoL (r = 0.50, p < 0.01). Daily activity from actigraphy did not predict symptom severity of non-motor and motor-related complications. Discussion: Pilot results show utility of actigraphic metrics for indexing real-world mobility and QoL declines in neurodegenerative disorders, in line with broader efforts to turn real-world data into actionable evidence for healthcare interventions. Ongoing discovery in larger populations should yield robust, clinically-relevant indices of daily activity in aging and neurodegenerative impairment.

